# Utilization of Over-the-Counter Painkillers Among Medical Students During Academic Examinations

**DOI:** 10.7759/cureus.43706

**Published:** 2023-08-18

**Authors:** Mohammad H Rajab, Shaza K Ewis, Kawthar Almatar, Lena Y Abdelmajed, Maryam S Ba Sowid, Maryam O Bajaber, Raghad Aljejakli, Suaad Bin Saeedu, Tasnim Abbad, Zahraa S Alsultan, Shamah M Shabi

**Affiliations:** 1 Biostatistics, Epidemiology, and Public Health, Alfaisal University College of Medicine, Riyadh, SAU; 2 Medicine, Alfaisal University College of Medicine, Riyadh, SAU

**Keywords:** otc painkillers, saudi arabia, alfaisal university, academic exams, medical students

## Abstract

The utilization of over-the-counter (OTC) painkillers among medical students during academic exams has raised concerns about health risks and potential implications, including substance abuse and academic performance. This cross-sectional study aimed to estimate the prevalence of OTC painkiller utilization among medical students at Alfaisal University during academic exams. Additionally, the study explored and identified the factors that influenced the patterns of OTC painkiller utilization among these students. The study was conducted from January to May 2023, following approval from the Institutional Review Board. The research involved surveying medical students of different genders, nationalities, and academic years during examination periods. Out of 1,500 medical students, 194 participated, resulting in a response rate of approximately 13%. The study results revealed that 50.5% of medical students used OTC painkillers during exams. While there were no significant variations based on gender or nationality, the prevalence of OTC painkiller utilization varied significantly across academic years.

During exam periods, the primary reason reported for using OTC painkillers was pain management. Additionally, some students mentioned using OTC painkillers to seek relaxation, improve sleep, enhance concentration, and stay alert. These insights shed light on the coping strategies practiced by students during high-stress academic periods. Most participants demonstrated familiarity with the guidelines for safe OTC painkiller utilization. Although the majority used painkillers infrequently or as needed, a subgroup reported increased utilization during exams. This finding highlights the importance of continuous monitoring and health education initiatives to prevent or address potential OTC painkiller abuse among medical students during exam periods. Ensuring the well-being of medical students is a priority, and appropriate measures should be employed to address any emerging concerns related to substance abuse. By addressing these issues proactively, educational institutions can promote a healthier academic environment during exam periods.

## Introduction

Over-the-counter (OTC) medications are drugs that individuals can legally purchase without a prescription [1]. Common examples of OTC medications include painkillers such as acetaminophen, ibuprofen, and aspirin [2]. While OTC medications are considered safe when used as recommended, it is important to acknowledge the potential risks associated with their usage [3]. These risks may involve incorrect self-diagnosis, improper dosage, potential addiction with prolonged use, and possible drug interactions [4]. One must be cautious with OTC medications as they can be easily misused due to their legal availability, affordability, and absence of age restrictions or purchase limits [5].

The widespread availability of OTC medications has led to insufficient awareness regarding their potential for misuse and dependence [6]. While OTC medications may appear harmless, their improper usage can result in addiction and detrimental health issues like memory loss, kidney failure, heart problems, and even fatalities [1]. Consequently, studies have been conducted to assess the prevalence of OTC medication utilization among various demographics, including medical students [7, 8, 9, 10, 11, 12].

In a study conducted in Spain, it was found that 78.9% of the 727 respondents consumed OTC medications [7]. Painkillers were the most frequently utilized OTC medications (49.1%) [7]. A study conducted in Germany reported a higher prevalence of OTC medication use in women (52%) compared to men (40.8%) [8]. A study in South Korea involving 345 patients revealed that 64% of respondents had utilized OTC medications, with antipyretics, analgesics, and anti-inflammatories being the most used (43.5%) [9]. As reported in another study, the reasons for OTC medication utilization included cold symptoms (28.1%), dyspepsia (22.6%), and pain management (19.4%) [10]. In a 2018 study conducted in the United Arab Emirates, 51% of participants reported using OTC medications, with women exhibiting a higher likelihood of OTC medication usage compared to men (55.1% and 47.2%, respectively) [10]. A research study on nursing students from a public university in Brazil revealed that 38.3% of the participants resorted to OTC medications for pain management [13]. The frequently used painkillers included dipyrone (59.2%), paracetamol (19.8%), and nonsteroidal anti-inflammatory drugs (NSAIDs) (13.1%).

Research conducted at Ayub Teaching Hospital, Women Medical College, International Medical College, and Frontier Medical College in Abbottabad, Pakistan, revealed a notable prevalence of OTC medication usage [11]. Out of the 300 subjects approached, 297 (99%) reported engaging in self-medication, with NSAIDs being the most used (74.3%). Fever (13.7%), cough (12%), and headache (12.3%) were the primary symptoms triggering OTC medication utilization. Furthermore, 18.7% of students resorted to self-medication for immediate relief, another 18.7% expressed confidence in their knowledge of suitable drugs to take, and 19.7% viewed it as a time-saving measure [11]. In another study conducted among medical students in Pakistan during the COVID-19 pandemic, a significant proportion of participants (83%) reported engaging in self-medication using OTC drugs [12]. The most utilized medications were paracetamol (65.2%) and multivitamins (56.0%). Furthermore, female students and those in their third year of medical studies demonstrated a higher tendency toward self-medication compared to other students [12].

Few studies have specifically investigated the utilization of OTC medications among medical students in Saudi Arabia. A study conducted at King Abdulaziz University highlighted alarmingly high rates of OTC medication usage among medical students and interns [14]. Out of the 504 participants, 75.2% reported self-medication in the six months preceding the study. Furthermore, the use of OTC analgesics was more prevalent among students in higher academic years. The study also indicated that among students who used OTC painkillers, 49.6% of them opted for NSAIDs [14]. Another study conducted at a public sector university in Dammam, Saudi Arabia, revealed that 55.1% of the 450 students surveyed engaged in self-medication with OTC drugs. Among these students, 64.4% reported rare instances of self-medication, while 12% engaged in frequent self-medication [15]. OTC painkillers were the most used, reported by 72.35% of the students, followed by antihistamines and cold/flu products, reported by 39.16% of the students [15].

A study conducted among undergraduate students at King Khalid University in Abha, Saudi Arabia, shed light on the reasons behind the usage of OTC drugs during academic examinations [16]. The study identified several factors, including headache (35.6%), pain management (21%), fever (16.6%), and cough (8.2%). OTC painkillers were the most frequently used (57.2%), followed by antipyretics (16.4%). Furthermore, students provided justifications for their OTC medication usage, with 87% believing that OTC medications were not hazardous, 85.3% stating that students possess the knowledge to self-treat, 80.3% using OTC medications due to their affordability and easy accessibility, and 80.1% considering it a time-saving measure [16]. 

The existing literature has highlighted the high prevalence of OTC medication usage among medical students. However, to the best of our knowledge, no study has specifically examined the utilization of OTC painkillers among medical students during academic exams in Saudi Arabia. The study aimed to estimate the prevalence of OTC painkiller utilization among medical students at Alfaisal University during academic exams. Additionally, the study explored and identified the factors that influence the patterns of OTC painkiller utilization among these students. By investigating the prevalence of painkiller utilization during exams and understanding the underlying factors, this research intended to provide valuable insights into the medication practices of medical students during exam periods.

## Materials and methods

After receiving approval from the university's Institutional Review Board (IRB-20174), the study team conducted a cross-sectional study at Alfaisal University from January to May 2023. Alfaisal University, founded in 2002, is a fully accredited, private, nonprofit university in Saudi Arabia with over 3,000 students representing more than 40 countries [17]. The study aimed to estimate the prevalence of OTC painkiller utilization among medical students at Alfaisal University during academic exams, i.e., midterms, finals, objective structured clinical examination (OSCE), etc. Additionally, the study explored and identified the factors that influence the patterns of OTC painkiller utilization among these students.

The target population for this study consisted of approximately 1,500 medical students enrolled in Alfaisal University's College of Medicine. Exclusion criteria included College of Medicine faculty and staff members, graduating medical students, students who had taken part in the pilot test of the study survey, and individuals who were under 18 years of age.

The study investigators created an anonymous, self-administered online survey using Google Forms®. The survey was specifically tailored for medical students, and participants were given explicit instructions and any required clarifications. The survey commenced with an introductory paragraph, informing participants about the study objectives, guaranteeing the anonymity of their responses, and emphasizing their freedom to decline participation or refuse to answer specific questions.

The survey comprised a blend of closed- and open-ended questions. Closed-ended questions included participants' demographics, OTC painkiller usage during academic exams, factors that potentially influenced OTC painkiller utilization patterns during college exams, and familiarity with guidelines for the safe usage of OTC painkillers. Open-ended questions allowed participants to provide additional comments. As part of the survey validation process, ten medical students pilot-tested the survey draft. The pilot test revealed that it takes approximately ten minutes to complete the survey.

To initiate the survey process, an introductory email was sent to the target population through the institutional email system. The introductory email informed the medical students about the study objectives and solicited their participation. The web link for the survey was then distributed to the target population through the institutional email system. Afterward, two additional follow-up reminders were emailed to the same group.

Demographic information, such as gender, nationality, and academic year, was collected for each study participant. The primary outcome variable, the prevalence of OTC painkiller use among medical students during exams, was also recorded. Considering a study design with a 5% margin of error and a 95% confidence level, the minimum required sample size was 306 respondents. Data utilized in this study are available upon request from the corresponding author.

Descriptive statistics, including frequencies, were computed to summarize the demographic variables and the prevalence of OTC painkiller use among medical students during exam periods. Bivariate analysis, specifically the chi-square test, was performed to explore associations between the prevalence of OTC painkiller utilization during exams and variables of interest, namely gender, year, and nationality. A significance level of 0.05 was employed to determine statistical significance. Data management and analysis were conducted using the jamovi statistical spreadsheet [The jamovi project (2023). jamovi (Version 2.3) (Computer Software)]

## Results

The primary objective of this study was to estimate the prevalence of OTC painkiller utilization among medical students at Alfaisal University during academic exams. Additionally, the study explored and identified the factors influencing the patterns of OTC painkiller utilization among medical students during exams. Out of the 1,500 medical students invited to participate, a total of 194 students responded, resulting in a response rate of approximately 13%. Among the respondents, 73% were female, and 79% were non-Saudis. In terms of the academic year, 29% were first-year medical students, 20% were second-year students, 24% were third-year students, 17% were fourth-year students, and 10% were in their fifth year. Table 1 provides a summary of the baseline characteristics of the survey participants. These characteristics served as the basis for further analysis in understanding the prevalence and patterns of OTC painkiller utilization among medical students during the demanding academic exam period.

**Table 1:** Baseline characteristics (n = 194)

	Frequency (%)
Gender	
Female	141 (73)
Male	53 (27)
Year	
First	56 (29)
Second	39 (20)
Third	47 (24)
Fourth	32 (17)
Fifth	20 (10)
Nationality	
Saudi	40 (21)
Non-Saudi	154 (79)

The study participants were surveyed regarding their usage of OTC painkillers during academic exam periods. The results revealed that 50.5% of medical students reported using OTC painkillers. The prevalence of OTC painkiller usage among medical students at Alfaisal University College of Medicine is presented in Table 2.

**Table 2:** Prevalence of OTC painkillers utilization (n = 194)

	Frequency (%)
OTC painkillers usage	98 (50.5)

The statistical analysis revealed that the prevalence of OTC painkiller usage did not exhibit a significant difference based on gender (p = 0.37) or nationality (p = 0.178). However, there was a significant variation in the prevalence of OTC painkiller usage across different academic years, with the highest frequency observed among fourth-year students. Table 3 provides an overview of the prevalence of OTC painkiller usage among medical students during exams, categorized by various characteristics.

**Table 3:** Prevalence of OTC painkiller utilization by various characteristics

Characteristic	Painkiller use Frequency	Painkiller use %	P-value
Gender			
Female	74	76	0.37
Male	24	24	
Nationality			
Saudi	24	24	0.178
Non-Saudi	74	76	
Year			
1^st^	17	30	0.007
2^nd^	24	62	
3^rd^	25	53	
4^th^	21	66	
5^th^	11	55	

The study participants were also inquired about their familiarity with the guidelines regarding the safe use of OTC painkillers. Among all respondents, 61% reported being acquainted with these guidelines. Most survey respondents stated using Acetaminophen, followed by nonsteroidal anti-inflammatory drugs (NSAIDs). Among the survey participants, 57% reported using OTC painkillers as needed, 16% reported occasional usage, and 9% reported rare usage. Table 4 presents the frequencies of the primary outcomes for OTC painkiller users. 

**Table 4:** Frequencies of the primary outcomes (n = 98)

	Frequency (%)
Familiarity with the guidelines for the safe use of OTC painkillers	60 (61)
Type of OTC painkillers	
Acetaminophen "Panadol”	80 (82)
Nonsteroidal anti-inflammatory drugs (NSAIDs)	10 (10)
Other	8 (8)
How often OTC painkillers were used	
As needed	56 (57)
Occasionally	16 (16)
Rarely	9 (9)
Other	17 (17)

The survey participants were also queried about their motivations for using OTC painkillers during academic exams and the factors that contributed to increased usage. The primary reported reason for OTC painkiller usage during exam periods was to alleviate pain. Other reasons included seeking relaxation, better sleep during exams, enhanced concentration, staying alert, and managing illness symptoms like flu. The factors identified for increasing OTC painkiller usage during exams were severe pain, familiarity with the effectiveness and safety of OTC painkillers, and the ease of accessibility to OTC medications.

## Discussion

Examinations exert significant stress on medical students, leading many to seek relief from pain or discomfort through OTC painkillers. The study reported that half of medical students were utilizing OTC painkillers during exam periods. This result agrees with other studies on similar populations [16]. The study findings showed that there were no statistically significant gender or nationality differences in the usage of OTC painkillers by medical students during their exams. In contrast to other studies focusing on similar populations, which reported a higher tendency among females toward self-medication [12, 18], the study results did not reveal such distinctions.

However, it is essential to acknowledge that significant variations in self-medication practices were prevalent among medical students in different academic years. This finding is consistent with other studies conducted on similar populations [14]. The observed variations by academic year highlight the relevance of continuous monitoring and health education initiatives. These initiatives should encourage responsible medication usage and promote healthier coping mechanisms for all students, regardless of gender or nationality.

Painkillers are among the frequently used drugs by college students [19]. The frequent use of OTC painkillers can lead to substance abuse, even with medications considered relatively safe when used as directed [19]. In this study, most participants did not report frequent usage of OTC painkillers. Most medical students reported using OTC painkillers only when needed during exams, or rarely. However, some participants mentioned an increase in OTC painkiller usage during exams. Factors contributing to this phenomenon included familiarity with the effectiveness and safety of OTC painkillers and the ease of accessibility to such medications. Additionally, severe pain during exams was identified as a factor for the more frequent use of OTC painkillers. Notably, substance abuse concerns were not reported.

Monitoring prevalence rates of painkiller usage is crucial for academic institutions to identify early warning signs and address potential substance abuse issues among medical students. While the overall prevalence of frequent OTC painkiller usage was not high in the study population, it is crucial to remain vigilant about potential risks. Healthcare professionals should continue monitoring painkiller usage patterns among medical students, particularly during high-stress periods like exams. Early identification of any concerning trends can help implement health education initiatives to promote responsible medication usage among medical students during exam periods. OTC painkillers are considered safe when used as recommended, but it's essential to acknowledge that they can still carry risks. Fortunately, most of the study participants reported being familiar with the guidelines for the safe use of OTC painkillers.

It was reported that certain OTC painkillers could impact a student's health or academic performance [20]. In this study, the primary reasons reported for OTC painkiller usage during exam periods included seeking relaxation, pain management, better sleep, enhanced concentration, staying alert, and managing illness symptoms like flu. Pain and discomfort can be distracting, making it difficult for students to concentrate and perform at their best [20, 21].

OTC painkillers are considered safe when used as recommended, but it's essential to acknowledge that they can still carry risks [22]. Fortunately, most of the study participants reported being familiar with the guidelines for the safe use of OTC painkillers. However, estimating the prevalence of painkiller usage helps identify potential adverse effects or complications associated with their use. Providing information on safe usage and potential risks of OTC painkillers helps students make informed decisions about painkiller usage during challenging academic periods. Recognizing the changing patterns of painkiller usage throughout a medical student's academic journey is critical in designing effective interventions to safeguard their well-being during demanding exam periods [22].

This study estimated the prevalence of OTC painkiller utilization among medical students at Alfaisal University during academic exams and identified factors that influence the patterns of OTC painkiller utilization during exams among these students. The study findings are expected to contribute to promoting students' well-being and may inform the development of targeted interventions and support mechanisms to ensure responsible and informed painkiller usage among this student population.

Survey studies possess inherent limitations that can influence the validity and generalizability of findings. A notable constraint in this study design was the presence of self-reporting bias, wherein respondents may have unintentionally or intentionally provided inaccurate or biased information. This bias can be influenced by recall bias, where individuals inaccurately recall past events or experiences. Moreover, the use of convenience sampling in this study introduced a sampling bias, potentially restricting the generalizability of the findings. It is important to note that certain groups, specifically females, were overrepresented, which could affect the validity of the conclusions drawn from the survey results [23]. 

The study initially aimed for a sample size of 305 participants; however, only 194 responses were received, suggesting the presence of non-response bias. Additionally, the survey utilized standardized questions with predetermined response options, limiting the depth of information gathered. Consequently, significant differences crucial for achieving accurate and thorough conclusions may have been overlooked. Moreover, the survey identified relationships between variables but did not establish causal relationships. Establishing causality necessitates experimental or other study designs [24].

## Conclusions

The study offers valuable insights into the use of OTC painkillers among medical students during exams. Approximately half of the students at this institution resorted to OTC painkillers to manage exam-related pain and stress. Interestingly, the reliance on these painkillers varied significantly across academic years, while gender and nationality showed no significant differences. The absence of significant gender and nationality differences suggests that painkiller utilization patterns remained relatively consistent among diverse medical student populations at this institution. Most study participants were well-versed with the guidelines for safe OTC painkiller use, supporting the notion that medical students are knowledgeable about responsible medication usage. During exam periods, the primary reason reported for using OTC painkillers was pain management. Additionally, some students mentioned using OTC painkillers to seek relaxation, improve sleep, enhance concentration, and stay alert. These motivations shed light on the coping strategies employed by students during high-stress academic periods. Although most participants reported using OTC painkillers infrequently or only when needed, a subgroup indicated higher usage during exams. This observation serves as an early warning sign for potential substance abuse issues among medical students, which require further attention and support. Ensuring the well-being of medical students should be a priority for the institution, and appropriate measures should be implemented to address any emerging concerns related to substance abuse.
